# The ART of Embryo Selection: A Review of Methods to Rank the Most Competent Embryo(s) for Transfer to Optimize IVF Success

**DOI:** 10.3390/biomedicines13112766

**Published:** 2025-11-12

**Authors:** Naiya Amin, Karen Kteily, Stacy Deniz, Mehrnoosh Faghih, Megan F. Karnis, Shilpa Amin, Michael S. Neal

**Affiliations:** 1 Department of Biology, McMaster University, Hamilton, ON L8S 4L8, Canada; 2 Ontario Network of Experts in Fertility (ONE Fertility), Burlington, ON L7N 3T1, Canadamneal@onefertility.com (M.S.N.); 3 Department of Obstetrics and Gynecology, Division of Reproductive Endocrinology and Infertility, McMaster University, Hamilton, ON L8S 4L8, Canada

**Keywords:** assisted reproductive technology, IVF, PGT, embryo selection, pregnancy

## Abstract

Within the field of assisted reproductive technologies (ARTs), embryologists regularly face the critical task of identifying embryos with the highest likelihood of implantation and survival. To help aid and standardize this practice, many embryo selection strategies have been developed to give the best chance of pregnancy success. Over the years, there has been a large increase in experimental studies conducted within this area of research. This increase has allowed for the formation of significant and plausible theories of embryo development, especially in cases where the most prominent factors seem identical. These advancements have both expanded the typical process of traditional treatments and have even paved the way for new techniques. The exact combination of all these relevant factors has not been fully elucidated into a single all-encompassing scheme for embryo decision. Morphological, genetic, and developmental indicators are well-studied individually, but the exact methods that should be prioritized in each scenario may change with respect to an individual patient. Deciding whether factors like age, egg quality, lifestyle choices, or previous medical history should alter methods of embryo ranking can result in conflict, especially in the case where a choice is being made between two similar embryos. This article reviews the conventional methods along with emerging technologies that provide the tools for embryologists to evaluate and rank embryos with high implantation potential (HIP). By showcasing these methods, including their respective benefits and drawbacks, this article provides information to allow clinicians to make effective decisions by integrating multiple approaches to embryo selection.

## 1. Introduction

This review emphasizes biological and embryological selection criteria rather than exploring external demographic or environmental influences, though there is some evidence to highlight these factors as a potential cause(s) of declining birthrates [1]. Embryo grading and progressive development timing will be observed in detail, especially regarding the cleavage and blastocyst stages. Different grading systems will be compared against each other to see which results in the best implantation and success rates. The need for a new, all-encompassing system will also be discussed [2]. Preimplantation genetic testing (PGT) to prevent both aneuploidy and other relevant gene defects has become another crucial procedure regarding many IVF cycles. Many techniques and subcategories of PGT have been explored to help further improve both pregnancy and live birth rate statistics [3]. Additionally, a combination of proteomics, metabolomics, and timelapse imaging to measure morphokinetics will be investigated to see their potential for future forms of analysis [4]. The rising importance of AI investigation as an emerging field will also be explored in the context of timelapse imaging. These factors will be evaluated to explore their current limitations and potential improvements. Additionally, any relevant clinical challenges or potential solutions will be discussed.

**Figure 1:**
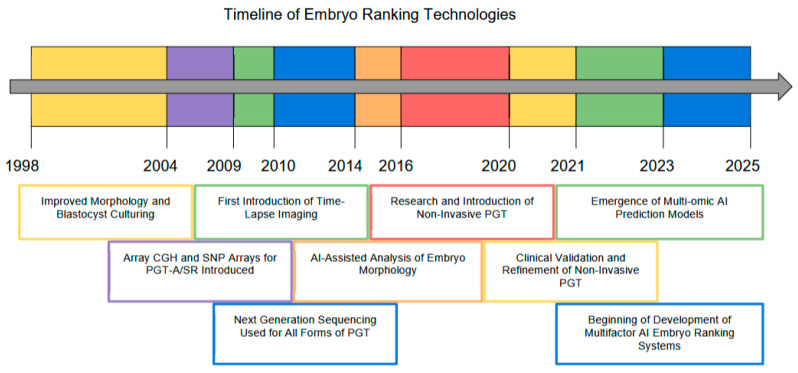
A timeline indicating the evolution of embryo selection strategies and development of related technologies.

This overview of embryological consideration provides insight into the many important facets of embryo selection in ART. The evolution of embryo selection strategies is outlined in Figure 1. This review addresses the future need to emphasize factors that are not regularly highlighted in embryo ranking, especially more abnormal clinical considerations. By recognizing the interplay between these factors, the groundwork for continued advancement in reproductive medicine can be expanded. In the near future, changes can be made to prioritize both scientific rigor and compassionate care for those seeking to build families.

## 2. Under-Recognized Clinical Considerations Impacting Implantation and Embryo Transfer Success

Implantation is a crucial step for pregnancy, whereby an embryo attaches to the uterine wall. Embryo implantation depends on several factors, including high embryo quality, proper timing and uterine receptivity, and hormonal and lifestyle factors (Table 1). One of the most significant limiting factors of all ART procedures is embryo aneuploidy resulting from maternal age. However, whether embryo ploidy status considers all aspects of age-related fertility decline remains controversial. Separate to ploidy status, abnormalities in the oocyte cytoplasm such as organelle dysfunction, lack of macromolecules, and altered gene expression have been shown to largely affect fertility outcomes [5]. These cytoplasmic factors may compromise embryo development even when chromosomal status appears normal. This is further supported by the increased live birth rate success of older patients when using young donor embryos [6]. Furthermore, as research has advanced, the role of the uterine environment in implantation and early development has become more apparent. In the past, the success of births from donor oocytes was directly indicative of a functional endometrium in older patients. However, more recently, it has been shown that declining endometrial gene expression can decrease fertility in patients above the age of 35 [7]. More specifically, the downregulation of ciliogenesis seems to be the leading cause of this deficiency [7]. A previous history of infertility also impacts the uterine microenvironment [5]. Despite the many negative outcomes of multiple gestation births at older ages, most patients opt to transfer multiple blastocysts despite the risks [8]. Medically, it has been shown that elective single embryo transfers are safer for both the mother and offspring [8]. Individualized treatment strategies should be considered in older patients to optimize outcomes while minimizing the unnecessary risks of multiple gestation pregnancies.

Male factors contribute to approximately one-third of infertility cases worldwide [9]. Age, out of many other individual factors, appears to be the most significant contributor and can contribute to poor semen quality, often resulting in oligo or azoospermia [10]. There is also mild evidence to show that paternal age impacts blastocyst development and euploidy rates [11]. After opting for oocyte donations, fathers of advanced age saw a relative decrease in embryo formation [12]. The most likely causes of this decrease are abnormal sperm morphology and genetics. There are many different criteria considered in modern day sperm analysis. The strict system categorizes sperm quality into three groups based on a histologic assessment of defects in the sperm head, neck, body, and tail [13,14]. The percentage of normal forms determines its prognosis. Additionally, the regulation of epigenetics is crucial to embryo development and can be significantly affected by sperm health [15]. Histone modifications and deoxyribonucleic acid (DNA) methylation through paternal ribonucleic acid (RNA) expression can have the potential to impact embryo viability [15]. Many forms of treatment for maladaptive male factors can be provided through ART [16]. Hormonal therapies, testicular sperm extraction, and intracytoplasmic sperm injection (ICSI) through IVF can all greatly improve fertilization, embryo development, and ultimately pregnancy success.

Different genetic conditions can affect gamete and embryo viability. In many cases, pregnancies involving either the monosomy or trisomy of various chromosomes rarely make it to term [17]. Testing sperm cell aneuploidy remains difficult and is only valuable as a diagnostic tool since there is no method available to determine the genetic composition of live sperm to be used for ICSI. As a result, normal embryo development is more heavily prioritized for ART [17]. IVF treatments can greatly aid both male and female patients that are known to be carriers of the gene associated with cystic fibrosis, both allowing for genetic counseling and increasing pregnancy to up to a 40% success rate [18]. Cystic fibrosis often affects the reproductive systems in both sexes, necessitating assisted reproductive technologies to achieve conception. Regarding both nutrient and medicine absorption, weighing the risks between fetal and maternal health is a crucial balance [18,19]. ART can also aid patients with classic galactosemia and its resulting primary ovarian insufficiency (POI) [20]. This inability to digest galactose largely affects important gonadotropin mechanisms and can greatly impair folliculogenesis even with early diagnosis and other treatments [21]. Structural chromosomal abnormalities including translocations or inversions are another possible cause of patient infertility. Most often, Robertsonian translocations present with a normal phenotype and are only detected through karyotyping [22]. These mutations significantly affect spermatogenesis and are heavily correlated with spontaneous miscarriages [23]. Additionally, Y chromosome microdeletions in sperm cells can lower euploidy rates [24]. Of the aneuploid embryos produced in this study, most saw the deletion or duplication of entire chromosomes [24]. However, in cases where euploid embryos had developed and been transferred, no differences were seen in clinical pregnancy rates [24]. These findings reinforce how subtle genetic alterations can have extreme effects on embryo quality and fertility outcomes. Knowledge of underlying chromosomal defects is an important component of comprehensive infertility assessments. This not only helps patients understand their infertility but helps clinicians develop a treatment plan with targeted therapies to maximize success.

Some other well-known clinical conditions, like polycystic ovarian syndrome (PCOS), can also negatively impact fertility outcomes by causing anovulation through hormone imbalance and the formation of multiple small antral follicles [25]. Letrozole therapy for patients with PCOS across multiple IVF cycles may improve their odds [26]. Proper assessments including cycle priming and supplemental vitamins or medicines should also be integrated into treatment plans [26]. Endometriosis is another prevalent infertility-related condition, and in severe cases, it may chronically affect a patient’s quality of life [27]. The disease itself may potentially hinder crucial steps involved in IVF, including ovarian response to gonadotropins and embryo implantation [28]. However, endometriosis has not been shown to negatively impact embryo aneuploidy or quality [28]. Other factors, such as uterine fibroids or blocked fallopian tubes, can act as physical barriers preventing pregnancy. Additionally, unhealthy weight and other lifestyle factors can have large impacts on fertility outcomes. Higher body mass index (BMI) ratios in women can result in a reduced number of oocytes from controlled ovarian stimulation and a need for a longer stimulation cycle [29]. In comparison to healthy individuals, overweight and obese patients were significantly less likely to achieve clinical pregnancy [29]. In males with higher body mass index ratios, testosterone levels were reduced, and semen was of poorer quality, though no effects on regular sperm parameters were observed [30]. Excess amounts of both physical and psychological stresses have been shown to reduce fertility in both genders, with physical stress having a more predominant effect on females than males [31]. Similarly, both illicit and prescription drug use, alcohol consumption, and smoking have all been correlated with patient infertility [31]. Environmental exposure to heavy metals and other chemicals can also negatively impact reproduction after secondary consumption [31]. It is unknown whether some of these components will become prominent screening factors for gamete viability. However, in certain contexts, they should be considered and spoken about to optimize live birth rate success when other evaluative components appear equal (Table 2). As a result, the promotion of a healthy lifestyle is foundational for infertility treatment.

## 3. Morphological Assessment

Various grading systems to assess normal embryological development have been developed. As advancements in research continue, assessment criteria may need to be altered to achieve the most desirable results. Underlying genetic or epigenetic variations may result in different embryo development rates. These differences have been strongly correlated with outcomes that underline the importance of developmental milestones. In addition to morphological markers, metabolic and epigenetic factors may need to be taken into consideration [32]. Creating a straightforward ranking system of embryo quality can be extremely complicated, with multiple facets to examine. Highlighting the most important features of development may instead provide a more well-rounded approach.

Evaluations of early-stage zygotes are usually conducted using pronuclear morphology, most specifically number, equality, size and distribution of nucleoli, pronuclear size and alignment, the time of pronuclear breakdown, and the presence or absence of a cytoplasmic halo [33]. Tesarik and Greco [34] first classified zygotes based on size and number as well as the distribution of nucleolar precursors to predict cell cycle progression. Their systems were later adapted and simplified. An additional method was proposed by Senn et al. [35] in 2005, where grades were assigned to six portions including proximity, orientation, and centering of the pronuclei, cytoplasmic halo, and number and polarization of nucleolar precursor bodies; these were then accumulated into a single score. More recently, there has been evidence to show that on average, smaller zygotes develop into higher quality blastocysts [36]. However, whether this distinction remains significant until implantation is unclear [37]. This suggests that while zygote size may be an early predictor of developmental potential, it must be interpreted cautiously within the broader embryological context.

High cell numbers, symmetrical blastomeres, and minimal fragmentation can also be great evaluative factors for embryos assessed at day three of development. Day 3 embryo transfers with six to eight blastomeres are most ideal [38]. Nomura et al. [39] showed that embryos with 7–8 blastomeres were more likely to develop to the blastocyst stage than those with <7 blastomeres on day 3. In the cases of these good quality embryos, earlier implantation seems to result in high clinical and ongoing pregnancy rates [38]. Additionally, it has been observed that lower microdrop volumes when culturing can result in higher day three cell numbers [40]. Whether or not a combination of these factors would contribute to higher pregnancy success rates is unclear. Though asymmetric blastomeres are heavily correlated with poorer implantation rates, pregnancy can still be achieved [41]. Often, this asymmetry is a sign of aneuploidy [41]. However, in cases where the embryo is still euploid and otherwise appears normal, it may reach the blastocyst stage of development [42]. In cases where asymmetry is the only real concern, it should not be used alone to justify selecting against the embryo for transfer and electing to discard. Similarly, day three fragmentation can also lead to successful birth in combination with other healthy factors, but there is some evidence showcasing that early fragmentation may hinder fetal development [43]. A lowered birth rate, preterm births, and other birth abnormalities are heavily correlated with fragmentation [43]. Day three embryo morphology is a crucial factor in ranking embryos for transfer and/or cryopreservation. More recently, with the trend toward extended culture, day 3 preimplantation embryo morphology is an important indicator for the potential to form a blastocyst. In cases where older patients are involved, it can even predict euploidy to prevent bad outcomes [44]. Continued evaluation of these factors is extremely helpful, especially when gross embryo morphology is not different.

Blastocyst development before transfer can also have significant insight into whether the embryo is competent enough to create a pregnancy that leads to a live birth. Across various forms of ART, including IVF, ICSI, and testicular sperm extraction ICSI, more profound blastocoel expansion and a larger blastocyst surface area have both been shown to result in ongoing pregnancy more often than smaller embryos and later developing blastocysts [45]. Similarly, a larger number of trophectoderm cells of good quality at this stage tends to promote better pregnancy results. These cells play a critical role in embryo implantation as the precursor cells that contribute to placental formation to facilitate implantation in the endometrium. The two factors, related by an osmotic gradient caused by a higher concentration of intracellular ions, allow the blastocyst to fill and expand appropriately [46]. Pluripotent stem cells in the center of the blastocyst, known as the inner cell mass (ICM), are key variables to consider when evaluating the implantation potential of blastocysts. Normal development of both the ICM and trophectoderm cells are extremely important morphological factors [47]. Morphokinetic progression shows that faster developing blastocysts (day 5) have higher implantation rates than their slower developing (day 6 or 7) counterparts, even with poorer morphological factors [48]. Ultimately, while morphology remains a valuable tool, developmental speed and cellular behavior may begin to offer powerful insight into an embryo’s potential for a successful pregnancy [49]. The Gardner grading system is a standardized method used in IVF to assess blastocyst quality based on three criteria, namely blastocoele expansion, ICM, and trophectoderm. The expansion stage is scored from one to six, indicating the degree of blastocoele expansion. A lower rating indicates an early and formative expansion, whereas a higher grade indicates more advanced growth [50]. The ICM, which forms the fetus, as well as the placenta-forming trophectoderm, are graded either A, B, or C to indicate cell amount, density, and quality [50]. Higher-quality blastocysts generally correlate with better implantation potential. Though this system is extremely reliable and easy to use, it has one weakness: deciding to implement between similarly graded embryos can be difficult. Utilizing other well-researched factors as well as knowledge of previous development rates can help patients make more well-informed decisions regarding their own treatments. It has been observed that blastocyst embryo transfer(s) as opposed to the cleavage stage embryos results in higher implantation success [51,52].

Embryo morphology is an extremely important criteria to consider for embryo selection. Though it cannot directly indicate the genetic normality of a particular species, the knowledge acquired over the years has allowed for a significant increase in pregnancy success. Additional research in this area has mitigated the need for multiple embryo transfers, mitigating some of the related dangers of multiple gestation pregnancy [53]. Continual improvement of morphological research will likely aid fertility practices all over the world.

## 4. Preimplantation Genetic Testing

Preimplantation genetic testing (PGT) is used during IVF cycles to screen embryos for genetic abnormalities before uterine transfer and to choose embryos with the best chances of having a healthy pregnancy. PGT can reduce the risk of transferring embryos with genetic anomalies, helping improve pregnancy success rates and decrease miscarriage risk. There are several variations in PGT, including (a) PGT for aneuploidy (PGT-A) to identify an unbalanced chromosome complement in the representative biopsied cells; (b) PGT for monosomic (PGT-M) gene disorders; (c) PGT for structural rearrangements (PGT-SR), and (d) more recently, PGT for polygenic (PGT-P) genetic traits.

PGT-A has consistently been used alongside morphological grading when making the decision to implant. Early PGT-A relied on a single cell biopsy from day 3 embryos and often failed to provide significant positive outcomes. The plausibility of preventing miscarriage through aneuploidy has promoted continuous research, and the advent of better culture media to support blastocyst development has led to trophectoderm biopsies instead of single cells. Along with advanced molecular techniques such as next-generation sequencing (NGS), this has prompted the technique to become more commonly employed in IVF labs around the world [54]. More recently, there have been opposing forces to both simplify the testing process and add greater acuity to the results [55]. Creation of a cost-effective, non-invasive, and a more widely available test could potentially allow more patients to receive this diagnostic test as part of their infertility treatment, though it may come with the sacrifice of data quality. Alternatively, the demand for higher genome resolution has also increased. These contrasting goals reflect the challenge of balancing accessibility with precision. The direction in which PGT-A testing will continue to develop is largely unknown, but the co-existence of these two streams with patient choice for utilization will likely result in the most overall satisfaction [55]. PGT-A testing is extremely helpful in preventing aneuploid transfer; however, about half of euploid transfers still may fail to implement [56]. There is some evidence to show that this is most largely correlated to low trophectoderm quality and late blastocyst expansion occurring on day six or seven [56]. Successful implantation requires proper timing and endometrial receptivity in addition to the transfer of an euploid embryo. Embryo selection based solely on chromosomal status may overlook other critical indicators of success. It is still unclear whether euploidy testing completely offsets the issues caused by older maternal age, especially considering the importance of non-chromosomal oocyte quality factors and acquired uterine factors [56]. Mosaicism can significantly affect PGT-A when testing is performed through trophectoderm biopsy [57]. This can result in both the disregard of low-level ICM mosaics as well as a selection of high-level ICM mosaics depending on which cells were biopsied and both the location and cause of aneuploidy. Since these mitotic errors can occur as early as day three of development, PGT-A testing may negatively affect mosaic outcomes [57]. Live births have resulted from embryos transferred after a mosaic diagnosis. This has fueled the debate about the value of PGT. Mosaics could be the result of a misdiagnosis, or perhaps the preimplantation embryo has the capability to correct or eliminate abnormal cells. Additionally, the need to use whole-genome amplification can result in “allele dropout and loss of heterozygosity in up to 25% of cases”, as well as any relevant laboratory errors [57]. Though PGT-A testing can provide much needed information to patients going through IVF cycles, the practice is not perfect, and other factors still occasionally affect pregnancy outcomes.

In cases where the patient or spouse has a known susceptibility to pass on genetic ailments to their children, PGT for monogenic conditions (-M) can be used to optimize both IVF success and quality of life for the future child. Though PGT-M predates PGT-A testing and usage [54], its use had not become extremely popularized until recently. This is plausibly because the two forms of PGT are most effective when used separately. For women under the age of 35, the usage of PGT-M without PGT-A is preferred to maximize successful results in cases where it is deemed necessary [58]. The implementation and routine usage of PGT-M often depend on prior linkage analysis and detailed family genetic histories, making it most applicable in cases where a known familial mutation has already been identified. Additionally, when used in combination with genetic counseling, PGT-M can remain an effective tool in ART for a variety of patients, including partners with a chromosome translocation or inversions and those using donor gametes [59]. After PGT-M, communication of poor test results to the family and understanding the relevant implications is an important part of the counseling process and can influence decisions made during future IVF cycles [59]. Some of the most common ailments targeted by PGT-M include cystic fibrosis, Tay–Sachs disease, thalassemia, and Huntington’s disease. The use of PGT-M, particularly with its detection of cancer predisposition syndromes, still faces some ethical challenges when considering hereditary conditions [60]. More specifically, the incomplete penetrance and variable expressivity of these syndromes raise questions about PGT-M legitimacy and necessity [61]. While PGT-M offers a powerful means of preventing many serious genetic conditions, its use must be carefully balanced with clear clinical guidelines. In cases where both parents are healthy, PGT-M should not be used as a form of genetic disease prevention.

PGT for structural re-arrangements (-SR) has emerged specifically as a targeted method for couples with chromosomal translocations, inversions, or duplications to prevent transmission of these changes to their children. This can be especially useful if the patients themselves carry balanced chromosomal rearrangements and appear externally asymptomatic [62]. Because of their altered genome, these individuals would have low fertility and could only naturally conceive children that would also be carriers [55]. Like most forms of PGT, PGT-SR is completed through next-generation sequencing of embryonic biopsies, rendering misdiagnosis possible dependent on the size and complexity of the re-arrangement [62]. Additionally, most PGT-SR are performed with standard PGT-A platforms if the affected regions are above the platform’s resolution. This is because embryos with unbalanced translocations can be identified by displaying segmental losses or gains in the regions involved in the translocation [55]. Deciding to utilize PGT-A, PGT-M, or PGT-SR, weighing the risks and benefits of miscarriage avoidance or reducing the chance of a healthy baby, will be a decision for each individual couple that should be informed by appropriate genetic counseling for their chromosomal rearrangement and history [63].

Similarly to PGT-M, PGT for polygenic conditions (-P) is another subset of PGT that focuses on screening for the risk of more genetically complex ailments. Recent advances have made genome-wide genotyping of IVF embryos feasible and affordable, potentially helping to screen for risk factors such as breast cancer, hypertension, diabetes, or schizophrenia [64]. Though PGT-P can reduce genetic risk factors for these ailments, environmental, lifestyle factors, or even random molecular events can still induce these diseases [64]. Its future use may begin to raise some ethical concerns, especially if technology starts being misused to screen for other genetic traits such as height or intelligence [65]. This could later lead to unnecessary usage of IVF amongst the population, potentially introducing otherwise inessential risks and harm [64]. Due to their similarities, it is important to consider the current regulations for PGT-M when creating healthcare regulations for PGT-P to be used in the field [65]. PGT-M and -P do not necessarily select HIP embryos but rather select against a genetic disease or trait. Therefore, they represent a different tool for the selection of embryo(s) for transfer and may be a valuable process for certain patients.

## 5. Non-Invasive Embryo Assessment

Time-lapse imaging (TLI) of embryos is a new non-invasive technique that requires special incubators with cameras incorporated within to take images of embryos every few minutes. With this technology, correlations between timing and stages of development, termed morphokinetics, are employed to create detailed algorithms for embryo selection. Specifically, it can highlight abnormal cleavage patterns, delayed divisions, or irregular morphologies associated with reduced viability [4]. Another bonus of TLI is that it removes the need to disturb or remove the embryos from the incubator for assessment [66]. This can also help the embryo stay within more regulated oxygen-concentrated environments, simulating the natural conditions of reproductive organs [4]. The KIDScore^™^ and early embryo viability assessment (Eeva^™^) algorithms are both proposed to aid in embryo choice after undergoing TLI. The KIDscore^™^ algorithm helps to differentiate between morphologically normal day 3 and day 5 embryos based on the presentation of abnormal cleavage patterns during their development, including slow and fast development [67]. These are also further compared and adjusted using Gardner’s grading system [67]. Alternatively, the Eeva^™^ test automatically measures cell division timings between first and second mitosis and between second and third mitosis to provide a high or low probability of blastocyst formation [68]. The Eeva^™^ system is indicated to provide adjunctive information on events occurring during the first two days of embryo development that may predict further development to the blastocyst stage on day five of embryo culture [68]. Despite its growing adoption, accessibility and cost may limit its widespread use among patients, especially as clinical guidelines still vary. As evidence for improved birth rates and reduced miscarriages is of relatively poor quality, some still argue that regular incubation infrastructure is more than sufficient [69] for most forms of ART. Whether or not this technology continues to improve itself in the future remains to be seen.

Non-invasive PGT (ni-PGT) is a new potential format for conducting PGT without needing to directly biopsy a cell sample. Through collecting blastocoele fluid of fully expanded blastocysts or by sampling spent culture media, free-floating DNA released from the ICM and trophectoderm cells can then be extracted and amplified to undergo genetic analysis, including PGT-A/M/SR [70]. The main advantages of ni-PGT include lowered potential for risk(s) associated with embryo biopsy, as well as reducing the cost of PGT treatment [70]. Furthermore, because the method relies on naturally secreted genetic material, it may offer a more accurate representation of the embryo’s overall genetic status compared to a representative cell biopsy. Additionally, the loss of blastocoele fluid should not, in theory, be detrimental to the embryo [70], as blastocoele collapse is routinely employed for vitrification and re-expansion of the cavity is frequently observed after warming.

Spent media can also be used to analyze protein profiles and metabolites, evaluating biochemical activity and molecular function. By identifying specific biomarkers through mass spectrometry, embryo viability, oocyte quality, implantation potential, developmental competence, or underlying reproductive disorders can all be recognized [71]. It is known that cells demonstrating higher pyruvate and glucose consumption through spent media also tend to showcase a higher likelihood of blastocyst formation [72]. On broader scales, metabolomics studies can also assist in evaluating the efficacy of infertility treatment drugs [73]. Determining the average gamete and embryo health of an individual and tracking changes in response to various treatments can give new insight into possible side effects and may further enhance live birth rates. Similarly, the identification of infertility-related proteins in spent media may give reason for implantation failure [73]. The metabolite 3-hydroxybutyric acid (3-HB) as well as the protein platelet endothelial cell adhesion molecule-1 (PECAM-1) both have new evidence to be implicated in female infertility without being related to other visible infertility disorders [73]. Whether or not these two will emerge as key biomarkers for implantation failure will rely on future research outcomes. If validated, their detection in routine spent media analyses could offer a non-invasive means of predicting reproductive success earlier in the IVF process. Using liquid chromatography–mass spectrometry, 3-HB can be separated and precisely quantified by its unique mass-to-charge ratio [74], while immunoaffinity enrichment coupled to targeted mass spectrometry enables the sensitive detection of low-abundance proteins such as PECAM-1 [75]. Recent large scale proteomic mapping of preimplantation embryos has revealed stage-specific proteomes varying between the morula, cleavage, and blastocyst stages [76]. Metabolic, mitochondrial, and junctional protein presence and function shift by large margins during early development, showing evidence for proteomic reprogramming that may heavily influence implantation potential during a short period of time [76]. A common limitation is the reproducibility of most metabolomic studies. As a direct consequence of the method, different samples of spent media need to be used for evaluation [72]. Depending on the cell, samples taken at different times may vary by large margins due to the complexity of cellular living [72]. Other potential biomarkers related to micro ribonucleic acids (miRNAs) include hsa-miR-661, hsa-miR-21–5p, and hsa-miR-372–5p. All three of these have limited evidence to show increased expression in degenerate embryos never reaching the blastocyst stage [77]. Information on current DNA-based biomarkers is limited. These have the potential to be used alongside protein and metabolic data to increase success with single embryo transfers. Integrating varied approaches could provide a more comprehensive and accurate assessment of embryo viability than relying on a single biomarker type alone.

The utilization of TLI alongside artificial intelligence (AI) programs opens new potential gateways involving ranking for single embryo transfer. At the 2019 meeting of the American Society for Reproductive Medicine, there were several abstracts that investigated the use of AI in analyzing raw TLI embryo development videos for non-invasive embryo selection. AI networks outperformed highly trained embryologists in selecting day 5 euploid blastocysts with high implantation potential [78]. This advantage is likely due to the computer’s ability to consistently recognize subtle, quantitative changes in morphokinetics that may be imperceptible to the human eye. By removing the variability introduced by human subjectivity, these systems can apply the same evaluation criteria across large datasets with unparalleled consistency. However, due to the relative infancy of this technology, standardized material for neural network training has not been introduced [79]. This has resulted in a variety of different neural networks coming to light. Additionally, depending on the training data network’s given some models can assess embryo morphology, while others may be more successful in predicting clinical pregnancy [79]. Most of these networks using convolutional neural network technology require a significant amount of computational power. However, they also establish the gateway for the creation of an AI program capable of combining TLI data as well as separating input clinical information [79]. This mixture of data types could eventually lead to the development of a singular evaluation entity, allowing for all necessary factors to be considered when making selection decisions in a systematic way that healthcare professionals may be unable to provide. The ethics behind such a development are still being debated [80]. Also, artificial intelligence analysis of TLI predicted that a selected embryo would spontaneously abort with 77% accuracy [78]. This significant success inspired the development of new neural network-based scoring models. Some examples, including BlastScoringNet [81] and STORK [82], have both seen success in their testing phases. Additionally, other algorithms focusing on different areas are still being created [82]. It is currently controversial whether images at critical development points or overall video evidence will be most helpful in predictive studies. It seems logical that videos would provide more information about embryo development, leading to better selection; however, individual critical images appear to be sufficient to precisely assess an embryo’s developmental competence [82]. Improvements in software analysis may leave this subject to change in the future.

## 6. Conclusions

The selection of embryos with the highest implantation potential remains one of the most complex steps in ART. While traditional approaches such as morphological grading and PGT have helped to improve pregnancy outcomes, their limitations indicate a progressive need to implement multifactorial strategies. For the best results, embryo selection will need to rely on an approach that accounts for biological factors while being adaptable to the needs of individual cases. Classic morphological grading using Gardner’s algorithm remains the most accessible and cost-effective method of assessing implantation potential. Improvements to incorporate both genetic and cytoplasmic factors as well as measuring cleavage-stage metrics and pronuclear morphology will aid in future rankings by allowing for a more holistic approach. Emerging non-invasive tools, including TLI, proteomic and metabolomic profiling, and artificial intelligence-driven analyses offer promising avenues to enhance precision without compromising embryo integrity. In removing the need for complete biopsy, embryos will be more likely to make it to term. However, the adoption of these techniques must be balanced against accessibility, cost, technical variability, and ethical considerations. The future of embryo selection will depend on combining established clinical knowledge with these innovations to create individualized, evidence-based protocols. In applying these changes, various fertility centers will be able to continue to support patients in achieving healthy, successful pregnancies.

## Figures and Tables

**Table 1:** Factors affecting embryo implantation for IVF-derived embryos.

Implantation Factor	Contributing Factors	Possible Clinical Treatment Solutions
Embryo Quality	Genetics—embryos with chromosome anomalies have a lower likelihood of implantation. Maternal Age—egg quality and increased chance of aneuploidy increase with female age. Sperm Quality—contributes genetic anomalies.	PGT-A is employed to identify euploid embryos. Advanced IVF culture systems and attention to detail in laboratories.
Endometrial Receptivity	Chronic endometritis—inflammation of the uterine lining interferes with implantation. Asherman’s syndrome–presence of scar tissue in the uterine lining. Insufficient uterine blood flow	Endometrial biopsy to detect inflammation or infections that can then be treated prior to embryo transfer. Promote endometrial lining thickness with hormone replacement therapy. Endometrial receptivity assays to determine optimal timing for embryo transfer.
Hormone Balance	An unbalanced hormonal milieux can interfere with the endometrial lining.	Monitoring of hormone levels (estradiol, progesterone) throughout the cycle. Supplementation with progesterone to promote endometrial lining growth or other hormones (thyroid stimulation hormone) as needed.
Immunological Factors	Immune response to a foreign object (ie: embryo) results in hindered embryo development and implantation.	Immunological testing to identify this issue Treat with immunoglobulins or corticosteroids to regulate an immune response
Lifestyle and General Health	Body Mass Index (BMI)—overweight and underweight conditions may impair implantation. Smoking, Alcohol, Drugs—affects gamete, embryo and endometrial quality.	Promote a healthy lifestyle for patients trying to conceive naturally or with assisted conception. Stress management (ie: mindfulness stress reduction).

**Table 2:** Summary table comparing different methods of embryo ranking and selection, including different advantages and limitations of each technique.

Technique (Most to Least Important)	When It Should Be Used	Advantages	Limitations	Criteria
Blastocyst Morphology/Morphokinetics	Whenever possible	-May be supplemented with AI analysis in the near future -Currently supported using non-invasive time-lapse imaging -Widely available/cost effective	-Grading may be subjective -No genetic assessment	-Garnder grading system (Good) -Day5/6 Blastulation
PGT-A/SR	Whenever possible, it should be more heavily considered for patients with advanced maternal age or recurrent implantation failure	-Help identify embryo- specific genetic abnormalities -Reduces implantation failure/miscarriage risks	-Invasive Biopsy -Can be expensive -Less effective for mosaic embryos	-Normal Result(s) on Day 5-6 trophectoderm biopsy
Ni-PGT	Whenever possible, especially in cases where biopsy has raised risks	-Non-invasive, Reduces risk of embryo damage -Complements morphological assessment	-Less accurate than traditional PGT, lower DNA quality -New, limited clinical adoption	-Normal Result(s) of spent media analysis
Pronuclear Morphology/ Morphokinetics	Whenever possible	-Currently supported using non-invasive time-lapse imaging -May help determine cleavage potential	-Predictive Value is limited, implantation potential may change through development	-Z-score system -High cell numbers -6-8 blastomeres
PGT-M/P	In patients who are known to either have or carry genes for genetic disorders	-Helps identify embryos impacted by hereditary disease	-Time consuming/expensive -Some ethical concerns regarding PGT-P testing	-Normal Result(s) on Day 5-6 trophectoderm biopsy

## Data Availability

No new data were created or analyzed in this study.

## Abbreviations

The following abbreviations are used in this manuscript:

AI	Artificial Intelligence
ART	Assisted Reproductive Technologies
IVF	In Vitro Fertilization
HIP	High Implantation Potential
PGT	Pre-implantation Genetic Testing
PGT-A	Pre-implantation Genetic Testing for Aneuploidy
PGT-M	Pre-implantation Genetic Testing for Monosomic Gene Disorders
PGT-SR	Pre-implantation Genetic Testing for Structural Rearrangements
PGT-P	Pre-implantation Genetic Testing for Polygenic Genetic Traits
Ni-PGT	Non-invasive Pre-implantation Genetic Testing
DNA	Deoxyribonucleic Acid
RNA	Ribonucleic Acid
ICSI	Intracytoplasmic Sperm Injection
POI	Primary Ovarian Insufficiency
PCOS	Polycystic Ovarian Syndrome
ICM	Inner Cell Mass
TLI	Timelapse Imaging
